# Identification of two distinct peptide-binding pockets in the SH3 domain of human mixed-lineage kinase 3

**DOI:** 10.1074/jbc.RA117.000262

**Published:** 2018-07-06

**Authors:** Malgorzata E. Kokoszka, Stefanie L. Kall, Sehar Khosla, Jennifer E. McGinnis, Arnon Lavie, Brian K. Kay

**Affiliations:** From the Departments of ‡ Biological Sciences and; § Biochemistry and Molecular Genetics, University of Illinois, Chicago, Illinois 60607

**Keywords:** phage display, protein-protein interaction, Src homology 3 domain (SH3 domain), ligand-binding protein, affinity selection, alanine scanning, combinatorial peptides, n-Src insert, mixed-lineage protein kinase, MLK3 SH3 domain-interacting peptide

## Abstract

Mixed-lineage kinase 3 (MLK3; also known as MAP3K11) is a Ser/Thr protein kinase widely expressed in normal and cancerous tissues, including brain, lung, liver, heart, and skeletal muscle tissues. Its Src homology 3 (SH3) domain has been implicated in MLK3 autoinhibition and interactions with other proteins, including those from viruses. The MLK3 SH3 domain contains a six-amino-acid insert corresponding to the n-Src insert, suggesting that MLK3 may bind additional peptides. Here, affinity selection of a phage-displayed combinatorial peptide library for MLK3's SH3 domain yielded a 13-mer peptide, designated “MLK3 SH3–interacting peptide” (MIP). Unlike most SH3 domain peptide ligands, MIP contained a single proline. The 1.2-Å crystal structure of the MIP-bound SH3 domain revealed that the peptide adopts a β-hairpin shape, and comparison with a 1.5-Å apo SH3 domain structure disclosed that the n-Src loop in SH3 undergoes an MIP-induced conformational change. A 1.5-Å structure of the MLK3 SH3 domain bound to a canonical proline-rich peptide from hepatitis C virus nonstructural 5A (NS5A) protein revealed that it and MIP bind the SH3 domain at two distinct sites, but biophysical analyses suggested that the two peptides compete with each other for SH3 binding. Moreover, SH3 domains of MLK1 and MLK4, but not MLK2, also bound MIP, suggesting that the MLK1–4 family may be differentially regulated through their SH3 domains. In summary, we have identified two distinct peptide-binding sites in the SH3 domain of MLK3, providing critical insights into mechanisms of ligand binding by the MLK family of kinases.

## Introduction

The human interactome has been estimated to contain ∼650,000 interactions (1) with many protein–protein interactions facilitated by modular domains (2–4). Among different techniques used to map the interaction of domains, phage display has proven successful at mapping their peptide binding preferences (5–8). This approach is based on the observation that peptides isolated through affinity selection of combinatorial peptide libraries usually bind to biologically relevant sites on the domain. Thus, it is possible to predict the candidate interacting partners of the domain in a particular target protein through a database search with the primary structure of the selected peptide ligands (9–12). Furthermore, the isolated peptides have the potential to serve as inhibitors or activators of specific cellular interactions in proof-of-principle experiments for drug development (13–15).

One of the most widely studied protein interaction modules is the Src homology 3 (SH3)^3^ domain (16–18). There are ∼300 SH3 domains in the human genome, present in over 200 proteins (19). Typically, SH3 domains bind proline-rich (P*XX*P) motifs (7, 20–22), and their specificity can often be readily revealed through examination of peptides affinity-selected from phage-displayed combinatorial peptide libraries (6, 7). However, several SH3 domains bind noncanonical motifs; for example, the Gads SH3 domain binds SLP-76 via an R*XX*K motif (24), the Eps8 SH3 binds e3b1/Abi-1 (25, 26) and RN-tre (27, 28) via a P*XX*DY motif (29), and the Fyn SH3 domain binds SKAP55 via an RK*XX*Y*XX*Y motif (30). Interestingly, structural studies show that most of the noncanonical ligand sequences are recognized by the same molecular surface of the SH3 domains (16, 17) that is used for binding proline-rich motifs.

A small number of SH3 domains, such as the Pex13p (31), p67^phox^ (32), and Vav (33) SH3 domains, bind noncanonical peptides via a different surface on the domain. More specifically, the SH3 domain of Pex13p (31) can independently bind two substrates with Pex14p interacting with the canonical site via P*XX*P motif and Pex5p interacting with a second noncanonical ligand-binding site in a proline-independent fashion. In the case of the p67^phox^ SH3–p47^phox^ interaction, both canonical and noncanonical binding surfaces facilitate interactions with respective P*XX*P and nonproline motifs present within the same p47^phox^-derived peptide sequence; this bivalent interaction results in a high-affinity and specific protein–protein interaction (32). Finally, the N-terminal SH3 domain of Vav cannot bind Pro-rich ligands because its RT loop (located between β1 and β2 strands of SH3 domain) contains a tetraproline insertion, which blocks accessibility of the canonical P*XX*P binding pocket; instead, it uses a different surface to bind the C-terminal SH3 domain of Grb2 (33).

Mixed-lineage protein kinase 3 (MLK3) is widely expressed in a variety of normal and cancerous tissues, such as brain, lung, liver, heart, breast, kidney, pancreas, and skeletal muscle (34–36). MLK3, also known as MAP3K11, is a serine/threonine protein kinase involved in regulating the JNK, p38, and ERK pathways (37–42). It is composed of several modules: an SH3 domain, a kinase domain, tandem leucine zippers, a Cdc42/Rac interactive binding (CRIB) motif, several linker regions, and a proline-rich C-terminal tail (39, 43). According to the BioGRID interaction database (44), MLK3 is involved in 36 unique interactions with several being mediated through the SH3 domain of MLK3. The SH3 domain has been implicated in the autoinhibition of the kinase domain through an intramolecular interaction via a nonconsensus single proline motif located between the leucine zipper and CRIB motif in the full-length MLK3 protein. Disruption of that intramolecular interaction results in increased activity of MLK3 (43). The SH3 domain of MLK3 also plays role in several other protein–protein interactions. The hepatitis C virus nonstructural 5A (HCV NS5A) protein was found to interact with the SH3 domain of MLK3 via a P*XX*P*X*R motif. Replacement of the two key prolines to alanine significantly decreases the interaction and NS5A's ability to block MLK3-induced apoptosis (45). The SH3 domain of MLK3 was also shown to interact with proline-rich regions in a hematopoietic progenitor kinase 1 (HPK1) (46, 47) and a germline center kinase (GCK) (42). Both kinases regulate the JNK pathway in an MLK3-dependent manner (42, 46).

Recently, MLK3 has been implicated in intracellular vesicle transport and motor neuron disease (48, 49), and given that the MLK3 SH3 domain contains a six-amino-acid insert, corresponding in position to the n-Src loop (50), we wondered whether the MLK3 SH3 domain might have novel binding properties. Here, we report the discovery of a novel noncanonical binding site on the SH3 domain of MLK3 that is topographically different from previous publications on noncanonical recognition by SH3 domains (31–33). The newly identified binding surface is composed of an extended n-Src loop (located between β2 and β3 strands) of the MLK3 SH3 domain, which opens up to bind a peptide ligand, named “MLK3 SH3–interacting peptide” (MIP), which lacks the canonical P*XX*P motif. Interestingly, when MIP binds to this site, it competes with binding of the HCV NS5A–derived peptide (P*XX*P*X*R) to the canonical binding pocket on the SH3 domain of MKL3. As the extended n-Src loop is conserved among the four members of the MLK1–4 protein family, we hypothesize that cellular interacting proteins exist that bind the SH3 domains of the MLK family of proteins in a unique MIP-like manner.

## Results

### MIP isolated from a phage-displayed combinatorial peptide library

**Figure 1.**
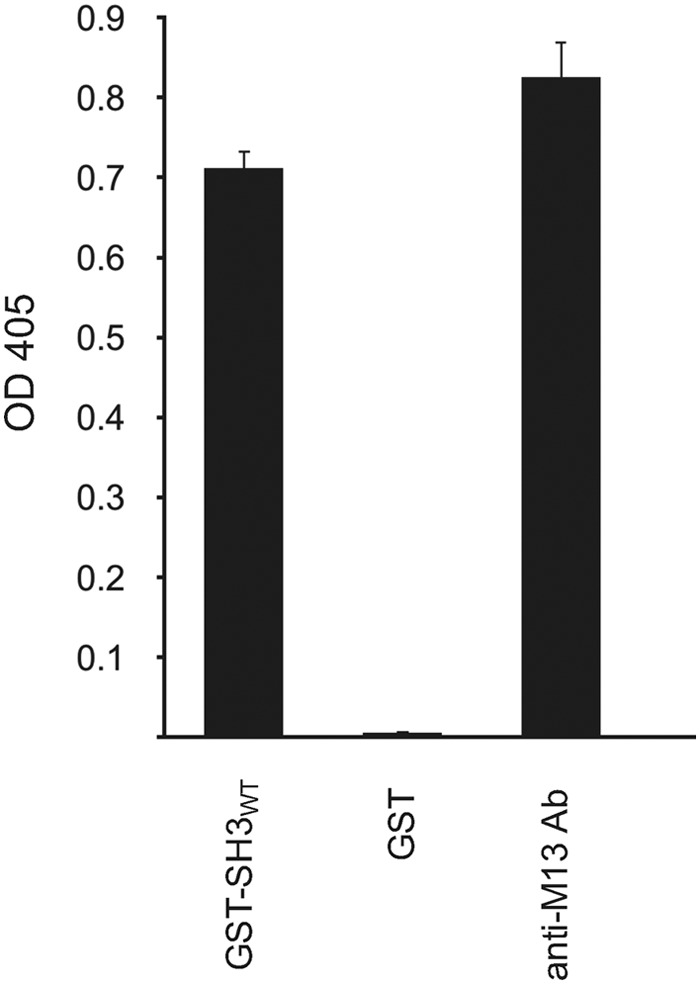
**Isolation of MIP from phage-displayed combinatorial peptide library.** Following three rounds of affinity selection with a phage-displayed combinatorial peptide library, ANL7 (51), a single peptide ligand, MIP (NH_2_-AIRINPNGTWSRQAETVES-COOH; insert (underlined) and flanking region), was identified to bind a GST-MLK3 SH3 domain fusion protein. ELISA shows the phage-displayed MIP to bind to the WT form of the MLK3 SH3 domain (SH3 WT) but not the GST fusion partner. The presence of virions in the supernatant was determined by coating the wells of the microtiter plate with an anti-M13 mAb (*anti-M13 Ab*). Virions, which were retained in the microtiter plate wells, were detected with anti-M13 mAb conjugated to HRP. Experiments were performed in duplicate, and the results are an averaged value; *error bars* reflect the standard deviation of each point. See also Fig. S1.

We utilized a GSH *S*-transferase (GST) fusion to the SH3 domain (amino acids 43–104) of human MLK3 (Fig. S1) for affinity selection of a phage-displayed combinatorial 12-mer peptide library (51). Following three rounds of affinity selection, we isolated a single clone displaying a 12-mer peptide, NH_2_-IRINPNGTWSRQ-COOH. As confirmed by phage ELISA, the clone bound to the wildtype (WT) form of the MLK3 SH3 domain (Fig. 1). Early experiments suggested that residues beyond the C terminus of the 12-mer peptide might contribute to binding, so we decided to characterize the binding properties of a 19-mer peptide, NH_2_-AIRINPNGTWSRQAETVES-COOH, named “MIP.” Truncation of MIP revealed that the entire 19-mer sequence is necessary for binding to GST-MLK3 SH3 (Fig. S2); for instance, deletion of Ala-1 (Fig. S2*A*) or deletion of several residues from the C terminus (Fig. S2*B*) resulted in lack of target binding.

### Alanine scanning of phage-displayed MIP identifies residues critical for binding and structure of the peptide

**Figure 2.**
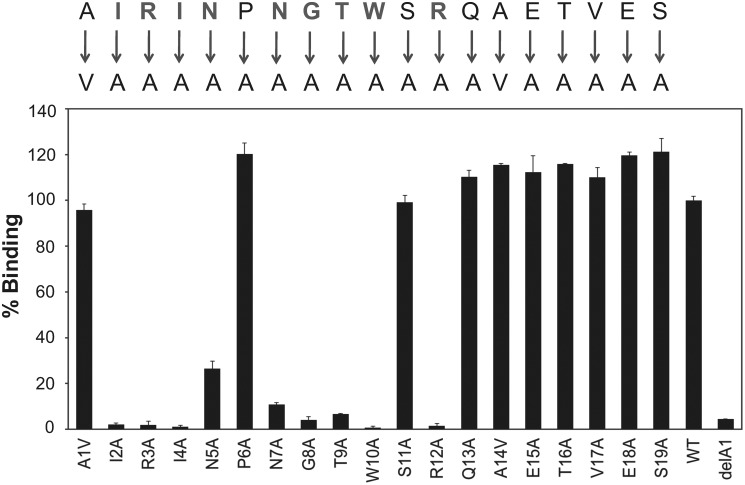
**Identification of residues critical for binding to MLK3 SH3 via alanine scanning of phage-displayed MIP.** The MIP sequence, NH_2_-AIRINPNGTWSRQAETVES-COOH, was fused to the N terminus of the pIII capsid protein of bacteriophage M13 (51) to facilitate type 3 pentavalent display. Systematic alanine replacements in the peptide were generated by Kunkel mutagenesis (11); Ala-1 and Ala-14 were replaced with Val. A truncated version of MIP (ΔA1) served as a negative control as deletion of Ala-1 resulted in loss of binding to MLK3 SH3. All phage-displayed variants were evaluated for their binding to GST-MLK3 SH3 via phage ELISA. The signal for binding of MIP (WT) to GST-MLK3 SH3 is set as 100%, and other values are normalized to it. Experiments were performed in triplicate, and the results are an averaged value; *error bars* reflect the standard deviation of each point. See also Fig. S2.

To determine which residues in MIP(1–19) are critical for binding, we performed systematic alanine replacement of the sequence and evaluated the variants in phage ELISA (Fig. 2). Alanine replacements at Ile-2, Arg-3, Ile-4, Asn-5, Asn-7, Gly-8, Thr-9, Trp-10, and Arg-12 were detrimental for the intermolecular interaction with GST-MLK3 SH3(43–104) (Fig. 2). Surprisingly, mutation of Pro-6 to Ala had no effect on binding (Fig. 2), indicating a noncanonical form of peptide binding to the MLK3 SH3 domain. Alanine replacements of Gln-13 through Ser-19 also had no effect on SH3 binding (Fig. 2); however, deletion of any of the C-terminal residues compromised binding to the MLK3 SH3 domain in the phage-displayed format (Fig. S2*B*) but not as a fusion protein (see Figs. 4 and S4). We interpret this as an indication that the C-terminal peptide region (amino acids 14–19) of MIP serves as a linker necessary to facilitate binding in the phage-displayed format.

### Properties of synthetic MIP

**Figure 3.**
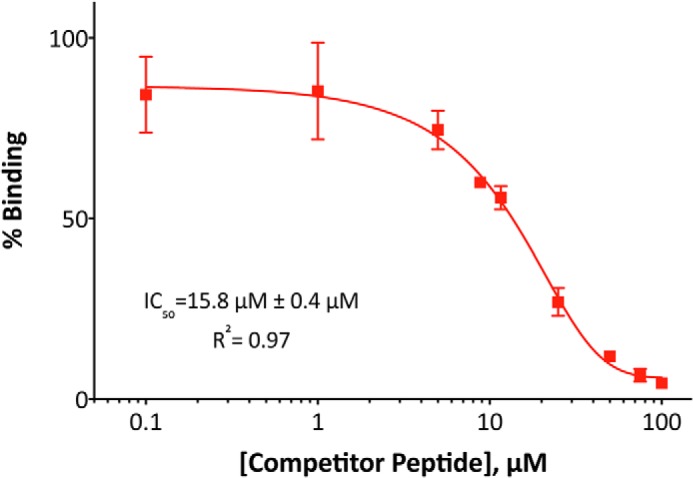
**Binding properties of synthetic MIP.** To determine the IC_50_ value of MIP, a GST-MLK3 SH3(43–104) domain fusion protein was preincubated with increasing concentration of unlabeled MIP (AIRINPNGTWSRQAETVES) as competitor and then allowed to interact with biotinylated MIP (AIRINPNGTWSRQAETVES-K-biotin) immobilized on a NeutrAvidin-coated 96-well ELISA plate. Binding of SH3 domain was detected with anti-GST antibody conjugated to HRP, and the levels are presented as percentage of binding in the absence of competitor. Experiments were performed in triplicate, and the results are an averaged value; *error bars* reflect the standard deviation of each point. The curve fitting was performed with GraphPad Prism 6.0 software.

Using competition ELISA, we determined the half-maximal inhibitory concentration (IC_50_) value of the MIP(1–19). In this assay, the GST-MLK3 SH3(43–104) protein was first incubated with increasing concentrations of unlabeled MIP (AIRINPNGTWSRQAETVES) as competitor and then introduced to a 96-well ELISA plate coated with biotinylated MIP (AIRINPNGTWSRQAETVES-K-biotin) captured via NeutrAvidin. The GST-MLK3 SH3(43–104) protein showed no cross-reactivity with NeutrAvidin or blocking agent; as anticipated, the GST tag showed no interactions with the MIP(1–19). The IC_50_ value for MIP binding the SH3 domain of MLK3 was observed to be 15.8 ± 0.4 μm (Fig. 3), which is in the range of binding affinities (*i.e.* single- to triple-digit μm) reported for multiple peptide–SH3 domain interactions (29, 52, 53).

### Architecture of the SH3 domain of MLK3

**Figure 4.**
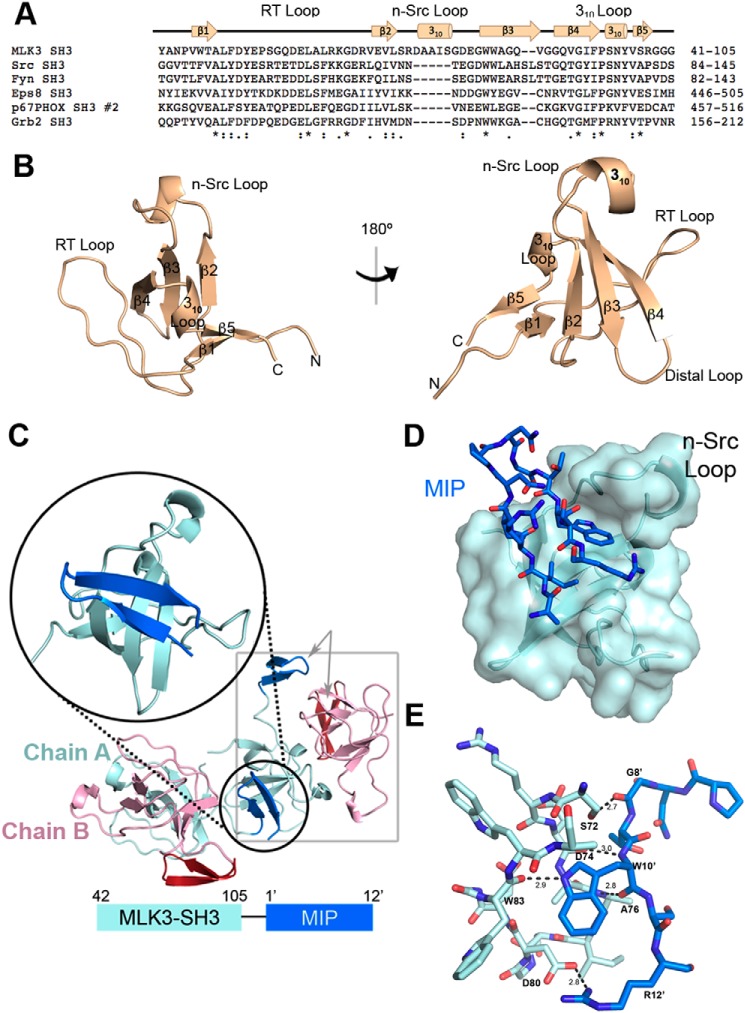
**The crystal structure of MLK3 SH3 illustrates the mode of binding of the phage display-generated MIP.** *A*, sequence alignment of the SH3 domains of human MLK3, Src, Fyn, Eps8, p67^phox^, and Grb2 was generated using Clustal Omega (23). Note the extended n-Src loop of MLK3 SH3. An *asterisk* indicates positions that have a single, fully conserved residue. A *colon* indicates conservation between groups of strongly similar properties, scoring > 0.5 in the Gonnet PAM 250 matrix. A *period* indicates conservation between groups of weakly similar properties, scoring ≤ 0.5 in the Gonnet PAM 250 matrix. *B*, pictured are two views of the ribbon-form structure of apo MLK3 SH3(44–103). The structure reveals a five-stranded antiparallel β-barrel flanked by the RT loop, n-Src loop, 3_10_ helix loop, and distal loop. A second 3_10_ helical structure is found in the extended n-Src loop. *C*, schematic of the crystal packing of MLK3 SH3 bound to MIP. Because the C terminus of MIP (13′–19′) had no clear electron density, it was not modeled. Although MLK3 SH3(41–105)-MIP(1–19) was expressed as a fusion protein, there was no intramolecular binding of MIP by SH3. Instead, the protein crystallized as a nonfunctional dimer with two chains in each unit cell (*gray box*). Chain A of the MLK3 SH3 dimer (*cyan*) binds the MIP from an adjacent Chain A (shown in *blue*; see zoom), whereas Chain B of the dimer (*pink*) binds the MIP (*red*) of an adjacent Chain B from a different symmetry mate. *D*, the MIP fits in a pocket formed by the n-Src loop of MLK3 SH3. Shown is the transparent surface rendering of MLK3 SH3 with a bound MIP. *E*, several residues are involved in the binding of MIP (*blue*) to the MLK3 SH3 domain (*cyan*). Hydrogen bonds formed by the interaction of MIP with MLK3 SH3 are denoted by *dashed lines* with distances in angstroms (Å). In particular, Trp-10′ and Arg-12′ are integral in forming several key hydrogen bonds. See also Figs. S3 and S4.

The sequence alignment of the SH3 domain of MLK3 and five other human SH3 domains (Src, Fyn, Eps8, p67^phox^, and Grb2) revealed that the n-Src loop sequence of MLK3 SH3 contains an elongated insert of five residues (Fig. 4*A*). We solved the X-ray structure of the apo MLK3 SH3(44–103) domain at 1.5-Å resolution and refined it to an *R*_work/_*R*_free_ of 18.6%/21.7% (Table 1). The final model has all residues in the allowed region of the Ramachandran plot. The overall structure reveals a five-stranded antiparallel β-barrel, a characteristic of all known SH3 domains, and three loops (RT, n-Src, and 3_10_ helix), commonly involved in the recognition of both proline-rich and nonproline ligands (16–18) (Fig. 4*B*). The residues that extend the MLK3 n-Src loop form a 3_10_ helix, which is not commonly present in SH3 domains (Fig. 4*B*).

**Table 1:** **Statistics for SH3–MIP complex and apo SH3**

Structure	MLK3 SH3–MIP	Apo MLK3 SH3	MLK3 SH3–NS5A
**PDB code**	5K26	5K28	6AQB
**Data collection**			
Space group	P3_1_	P3_2_21	C2
Cell dimensions			
*a*, *b*, *c* (Å)	64.01, 64.01, 35.20	57.89, 57.89, 76.12	80.63, 46.06, 49.89
α, β, γ (^o^)	90, 90, 120	90, 90, 120	90, 120.006, 90
Resolution (Å)	54.43–1.20 (1.27–1.20)*^a^*	50.14–1.49 (1.58–1.49)	40.26–1.50 (1.59–1.50)
*R*_merge_ (%)	3.9 (41.8)	7.6 (37.5)	3.6 (37.6)
*R*_meas_ (%)	5.3 (53.0)	8.4 (41.6)	4.7 (44.8)
CC1/2*^b^*	99.8 (75.7)	99.7 (89.0)	99.8 (85.9)
*I*/σ*I*	19.5 (2.8)	11.2 (2.9)	9.9 (1.6)
Completeness (%)	97.2 (83.1)	97.9 (95.6)	90.9 (86.9)
Redundancy	5.3	5.3	1.8

**Refinement**			
Resolution (Å)	1.20	1.50	1.50
No. of reflections	49,088	23,286	81,831
*R*_work_/*R*_free_ (%)	13.6/15.8	18.6/21.7	18.8/22.8
No. atoms			
Protein	Chain A: 583	Chain A: 508	Chain A: 530
Phosphate	5		5
Water	112	145	99
r.m.s.d.			
Bond length (Å)	0.016	0.022	0.022
Bond angles (°)	1.683	2.042	2.202
Ramachandran plot (%)			
Most favored*^c^*	95.7	94.9	100.0
Additionally allowed	4.3	5.1	0.0
Generously allowed	0	0	0
Disallowed	0	0	0

*^a^* Values in parentheses represent the highest resolution shell.

*^b^* CC 1/2 is a value for determining the data quality. Its value here is in %.

*^c^* From PROCHECK (63).

### The structure of MLK3 SH3–MIP complex reveals a novel binding site on the SH3 domain

To determine the structure of the MLK3 SH3–MIP complex, we constructed a fusion protein MLK3 SH3(41–105)-MIP(1′–19′) where the prime denotes residues of the peptide. This construct was expressed in bacteria and purified as described under “Experimental procedures.” Because the fusion protein elutes as a higher order oligomer from a gel-filtration column (Fig. S3), we expected the interaction to occur between two separate molecules of SH3-peptide fusion rather than an intramolecular interaction. The structure of MLK3 SH3(41–105)-MIP(1′–19′) was solved to 1.2-Å resolution and refined to an *R*_work/_*R*_free_ of 13.6%/15.8% (Table 1). The final model has all residues in the allowed region of the Ramachandran plot. As shown in Fig. 4*C*, the crystallographic asymmetric unit (*gray box*) contains two protomers that are labeled Chain A (*cyan*, domain; *blue*, peptide) and Chain B (*pink*, domain; *red*, peptide). The interaction of the SH3 domain of MLK3 with MIP occurs between two protomers that are related by crystallographic symmetry. The first residue in the SH3 sequence (Tyr-41) and the C terminus of MIP (13′–19′) have no clear electron density and, therefore, were not modeled.

The MIP binds in a pocket formed between the n-Src loop and β-strands 2–4 (Fig. 4*D*). As shown in Fig. 4*E*, multiple residues are involved in the binding of MIP to the SH3 domain of MLK3. Most notably, several hydrogen bonds contact Trp-10′ in the MIP sequence. The backbone amide group of Trp-10′ interacts with Asp-74, and the carbonyl group interacts with the backbone amide of Ala-76. Notably, both Asp-74 and Ala-76 are found within the MLK3-unique insert in n-Src loop of the SH3 domain. The Trp-10′ indole nitrogen atom hydrogen bonds with the carbonyl group of Trp-83 located on the edge of β3 strand. Gly-8′ and Arg-12′ also contribute to hydrogen bonding; the carbonyl group of Gly-8′ interacts with the hydroxyl group of Ser-72 in the SH3 domain, and the guanidine moiety of Arg-12′ forms a salt bridge with the side-chain carboxylate of Asp-80.

### Trp-10 and Arg-12 in MIP are critical for the intermolecular interaction with the MLK3 SH3 domain

To evaluate the importance of MIP residues Trp-10′ and Arg-12′ for binding to the MLK3 SH3 domain, we generated three fusion proteins composed of the SH3 domain linked via its C terminus to MIP(1–13)W10A, MIP(1–13)R12A, and the truncated version of the WT ligand, MIP(1–13). These purified proteins were analyzed by size-exclusion chromatography to determine their oligomeric state. Although the WT construct MLK3 SH3(41–105)-MIP(1–13) eluted as a higher order oligomer, both mutant constructs elute at a volume corresponding to the size of a monomeric fusion protein (Fig. S4). Mutation of either Trp-10′ or Arg-12′ to Ala resulted in a loss of binding to MLK3 SH3 (Fig. S4), consistent with alanine scanning results of phage-displayed MIP (Fig. 2).

### Comparison of the apo and MIP-bound MLK3 SH3 structures reveals closed and open conformations of the n-Src loop modulated by the ligand

**Figure 5.**
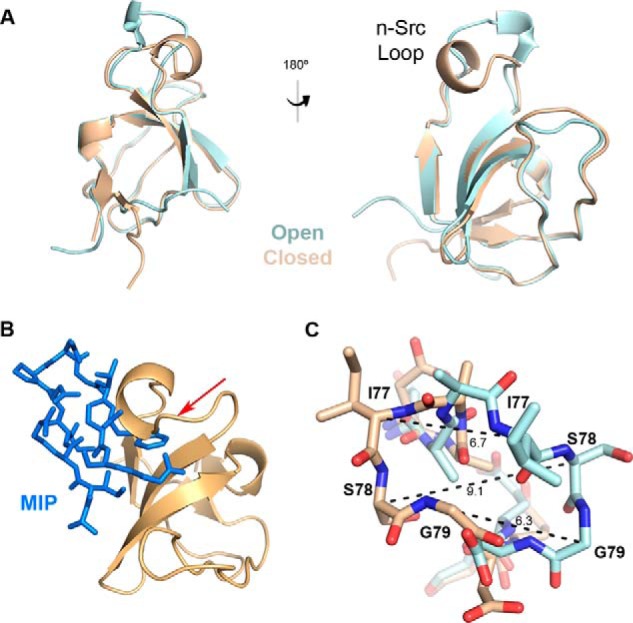
**Conformational changes in the n-Src loop induced by MIP binding.** *A*, crystal structure of apo MLK3 SH3(44–103) (*tan*) overlaid with MIP-bound MLK3 SH3(41–105) domain (*cyan*) shows a dramatic shift in the n-Src loop region, whereas the structure of the remainder of the domain is relatively unchanged. *B*, the n-Src loop of the unbound form of MLK3 SH3 must open up to allow MIP to bind the SH3 domain. Without this conformational change, MIP (*blue*) would not fit into the domain as evidenced by the clash indicated by the *red arrow. C*, a close-up of residues Ile-77, Ser-78, and Gly-79 in the n-Src loop in the bound (*cyan*) *versus* unbound (*tan*) structures. Measurements (Å) of the change of positions of the α-carbons in these residues demonstrate the spatial movement required to facilitate the interaction with MIP.

Comparison of the unbound and ligand-bound structures of MLK3 SH3 domain revealed a closed and open conformation of the n-Src loop, respectively (Fig. 5*A*). As modeled in Fig. 5*B*, the closed conformation of the loop precludes the peptide from the groove. To accommodate MIP as described previously, the n-Src loop undergoes a dramatic conformational change. In particular, residues Ile-77 and Ser-78, which participate in the 3_10_-helical structure, as well as the following Gly-79 undergo dramatic spatial movements required to facilitate binding of MIP to the SH3 domain of MLK3 as demonstrated by the change of positions of the α-carbons in these residues (Fig. 5*C*). The rest of the SH3 structure does not undergo any significant conformational changes upon binding with an r.m.s.d. of 0.41 over 40 residues (Fig. 5*A*).

### MIP binds to a noncanonical binding site on the SH3 domain of MLK3 in proline-independent manner

**Figure 6.**
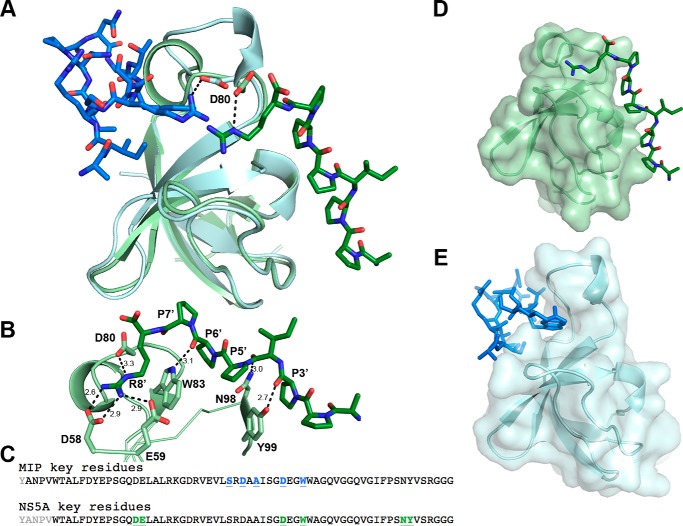
**Novel noncanonical binding pocket on the SH3 domain of MLK3.** *A*, an overlay of the SH3 domain from MLK3 (*cyan*) bound to MIP (*blue*) *versus* the SH3 domain from MLK3 (*light green*) bound to a canonical proline-rich peptide (*green*) derived from HCV NS5A. *B*, the NS5A peptide (APPIPPPR) interacts with several residues of the SH3 domain via hydrogen bonds, including Asp-58, Glu-59, Asp-80, Trp-83, Asn-98, and Tyr-99. The NS5A peptide makes contacts with the canonical binding pocket composed of the n-Src loop, RT loop, and 310 helix loop. *C*, the alignment of the sequences of MLK3 SH3 with the key interacting residues highlighted. Residues in *black* appear in the structure; key residues for MIP binding are shown in *blue underlined* text, whereas those crucial for NS5A binding are shown in *green underlined* text. Note that although Asp-80 and Trp-83 are involved in binding for both peptides only the Asp-80 side chain is shared between both structures. *D*, shown is the transparent surface model of MLK3 bound to NS5A. *E*, shown is the transparent surface model of SH3 domain of MLK3 bound to MIP in the same orientation as in *D*. MIP interacts with a noncanonical pocket formed by the extended n-Src loop, located on the opposite face of the SH3 molecule as compared with the canonical pocket. Note the change in orientation of the n-Src loop (top of domain).

Based on our structure, MIP fits into a novel binding site facilitated by a single n-Src loop and the edge of the β3 strand (Fig. 4*D*). In contrast, interactions with the canonical binding site commonly require involvement of three loops: RT loop, n-Src loop, and 3_10_ helix loop (20, 24, 31). The SH3 domain of MLK3 was previously reported to interact with the HCV NS5A protein (UniProt entry W8GG88) (45). The authors mapped the interaction to a P*XX*P*X*R motif, KA**P**TP**P**P**R**RRR (aa 2320–2331), present at the C terminus of the NS5A protein. To learn whether this peptide binds a site distinct from MIP, we fused the NS5A motif, AP**P**IP**P**P**R** from a different HCV genotype (UniProt entry A0A076MER2; aa 2322–2333) previously crystallized with the SH3 domain of c-Src (Protein Data Bank (PDB) code 4QT7), to the SH3 domain of MLK3 and prepared crystals for X-ray diffraction. The crystals diffracted to 1.5 Å with an *R*_work_/*R*_free_ of 18.8%/22.8%. Fig. 6*A* shows the overlay of the NS5A-bound structure compared with the MIP-bound structure. The NS5A sequence crystallized here is from a different genotype of HCV but carries the same P*XX*P*X*R motif that is conserved across all HCV genotypes (54, 55). Alignment of both complexes (Fig. 6*A*) shows that the two binding sites are on the opposite faces of the SH3 domain.

The NS5A peptide interacts with the canonical polyproline-binding site (Fig. 6*D*) by forming contacts with residues Asp-58 and Glu-59 of the RT loop, residues Asn-98 and Tyr-99 of the 3_10_ helix, residues Asp-80 of the n-Src loop, and residue Trp-83 of the β3 strand from MLK3 SH3 (Fig. 6, *B* and *C*). Conversely, MIP fits into a novel binding site defined by the n-Src loop and the edge of the β3 strand (Figs. 6*E* and 4*D*).

Interestingly, Asp-80 and Trp-83 of the MLK3 SH3 domain participate in binding both peptides; however, in the case of Trp-83, the side chain is implicated in interactions with the NS5A peptide (Fig. 6*B*), whereas its backbone carbonyl interacts with MIP (Fig. 4*E*). The Asp-80 side chain is the only one involved in both interactions (Figs. 6, *A* and *B*, and 4*E*); it is oriented differently depending on which peptide is bound and interacts with arginine residues present in both peptides (Fig. 6*A*).

### MIP and NS5A peptide bind the SH3 domain of MLK3 in competitive fashion

**Figure 7.**
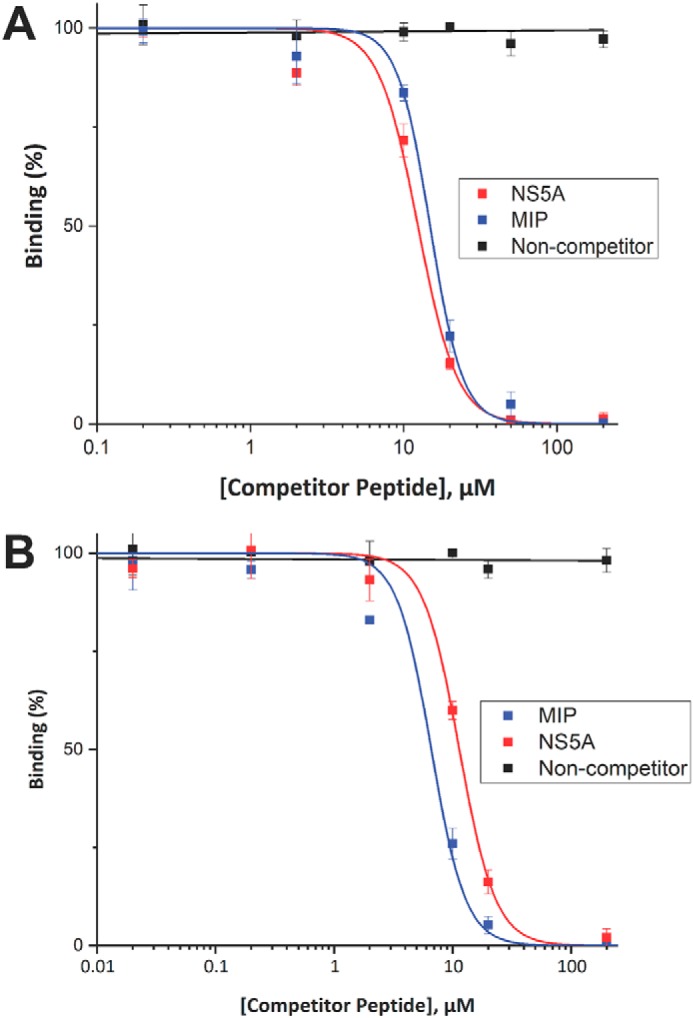
**Competition ELISA between MIP and NS5A peptide.** To determine whether the MIP and NS5A peptides competitively bind the SH3 domain of MLK3, a GST-MLK3 SH3(43–104) fusion protein was preincubated with increasing concentrations of unlabeled MIP peptide or unlabeled NS5A peptide as competitor and then allowed to interact with biotinylated MIP (*A*) or biotinylated NS5A (*B*) peptide probes immobilized on a NeutrAvidin-coated 96-well ELISA plate. Binding of the SH3 domain was detected with an anti-GST antibody conjugated to HRP, and the signal levels are presented as a percentage of binding in the absence of competitor. Experiments were performed in triplicate, and the results are an averaged value; *error bars* reflect the standard deviation of each point. Curve fitting was performed with OriginPro 2017.

Because Asp-80 in the SH3 domain of MLK3 participates in binding both peptide ligands, competition experiments were performed to determine whether MIP and NS5A peptide can bind the SH3 domain in an exclusive or simultaneous manner. The GST-MLK3 SH3(43–104) protein was first incubated with increasing concentrations of soluble MIP peptide (AIRINPNGTWSRQAETVES) or NS5A peptide (KKAPTPPPRRRR-GGG-K) and then transferred to a 96-well microtiter plate coated with biotinylated MIP peptide (Fig. 7*A*) or biotinylated NS5A peptide (Fig. 7*B*), which was captured on the microtiter plate well surface via immobilized NeutrAvidin. It was seen that the free NS5A peptide competes with immobilized MIP for binding to GST-MLK3 SH3(43–104) with an IC_50_ value of 12.9 ± 0.6 μm and that the free MIP peptide competes with immobilized NS5A for binding GST-MLK3 SH3 with an IC_50_ value of 6.6 ± 1.6 μm. This supports the crystallographic data in which Arg-12′ of MIP and Arg-8′ of NS5A would suffer a steric clash if bound at the same time as they induce a different side chain orientation in Asp-80 of the MLK3 SH3 domain (Fig. 6*A*). Additionally, previously described experiments noted the importance of Arg-12′ in the MIP peptide for overall binding (Fig. S4).

### Binding of MIP is conserved across most of the SH3 domains of MLKs

**Figure 8.**
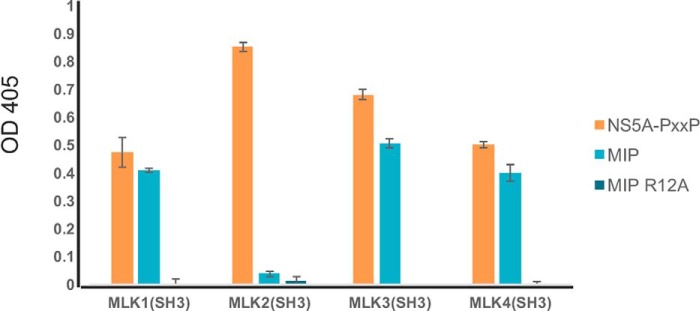
**Binding of MIP and NS5A peptides to SH3 domain of other members of MLK subfamily (MLK1–4).** To determine whether MIP and NS5A can interact with SH3 domains of MLK1–4 proteins, the purified GST-MLK1–4 SH3 domains were incubated with biotinylated peptides, NS5A peptide (KKAPTPPPRRRR-GGG-K-biotin), MIP (AIRINPNGTWSRQAETVES-K-biotin), and MIP-R12A (AIRINPNGTWSAQAETVES-K-biotin), immobilized on a NeutrAvidin-coated 96-well ELISA plate. Binding of SH3 domain was detected with anti-GST antibody conjugated to HRP. Experiments were performed in triplicate, and the results are an averaged value; *error bars* reflect the standard deviation of each point. See also Fig. S6.

MLK3 is one of the four members of the MLK subfamily (MLK1–4) (39, 56). To predict the likelihood of MIP cross-reacting with the rest of the subfamily, we generated protein homology models for SH3 domains of MLK1, MLK2, and MLK4 using the solved structure of MLK3 SH3 domain bound to MIP as a template (Fig. S6*A*). Although the primary sequences of the SH3 domains of MLK1, -2, and -4 are ∼60% identical to that of MLK3 (MLK1, 60.0%; MLK2, 66.15%; MLK4, 60.0%) (Fig. S6*B*), structurally the domains are predicted to be very similar based on Swiss-Prot modeling (Fig. S6*A*). Notably, four key residues involved in binding of MIP (Ser-72, Asp-74, Asp-80, and Trp-83) are conserved (Fig. S6*B*), and the overall predicted 3D structure of the n-Src loop and the rest of the molecule is highly preserved (Fig. S6*A*). To evaluate whether MIP and NS5A peptides can interact with SH3 domains of MLK1, MLK2, and MLK4, we constructed and purified GST fusions to each of these SH3 domains and examined their binding by ELISA. As can be seen in Fig. 8, the NS5A peptide could bind the GST-SH3 domain fusions of all (MLK1–4) purified proteins, whereas MIP bound the SH3 domains of MLK1, MLK3, and MLK4. Interestingly, the MIP peptide did not bind the SH3 domain of MLK2; comparison of the primary structures of MLK2 SH3 with the rest of the SH3 domains of the MLK subfamily (Fig. S6*B*) revealed a cysteine instead of a serine (MLK1) or alanine (MLK3 and MLK4) in the n-Src loop.

## Discussion

To our knowledge, this is the first report presenting insights into mechanisms of ligand binding by the SH3 domain of MLK3. Previously, a crystal structure of another member of the MLK subfamily, the SH3 domain of MLK2 (PDB code 2RF0), was determined. However, no structure of an SH3–ligand complex has been available until now. Our studies revealed a noncanonical binding site on the surface of MLK3 SH3 that is localized at the opposite face of the molecule compared with the site that accepts proline-rich motifs (Fig. 6). An examination of the crystal structures of several SH3 domains bound to noncanonical peptide ligands revealed that all bind the same side of the SH3 domain as do canonical proline-rich peptides (18), suggesting that the MLK SH3 domain has a novel binding site primarily defined by the extended n-Src loop (Fig. 4). In addition, as the key residues involved in the interaction of the SH3 domain with the MIP ligand are conserved across MLK1–4 (Fig. S6B), we suspected this noncanonical mode of SH3–peptide interaction to be potentially conserved across the other members of the MLK subfamily. Indeed, we found that MIP binds the SH3 domains of MLK1, MLK3, and MLK4 but not of MLK2 (Fig. 8).

In this study, we also mapped the location of the canonical binding site on MLK3 SH3 by solving a structure of the SH3 domain of MLK3 bound to the NS5A-derived peptide (APPIPPPR). Superposition of these two structures (Fig. 6) showed that the canonical P*XX*P and MIP-binding site are located at opposite faces of the molecule. However, based on the same overlay in Fig. 6, we also hypothesize that certain residues on the SH3 domain of MLK3 could be shared between those two different binding sites. MIP interaction with MLK3 SH3 requires involvement of Ser-72, Asp-74, Asp-80, and Trp-83 (Fig. 6*C* and 4*E*). Conversely, interaction of NS5A peptide with MLK3 SH3 seems to be dependent on Asp-58, Glu-59, Asp-80, Trp-83, Asn-98, and Tyr-99 (Fig. 6, *B* and *C*). Although the Trp-83 residue interactions are different for each peptide, the Asp-80 side chain is involved in interactions with arginines on both peptides that orient the side chain differently depending on the peptide bound (Fig. 6*A*). As both peptides require the carboxyl side chain of Asp-80 for binding, they could interact with the SH3 domain of MLK3 in competitive fashion as confirmed by competitive ELISA (Fig. 7). Additionally, mutation of a residue conserved among SH3 domains, Y52A, disrupts binding of the MLK3 SH3 domain to the MIP and NS5A peptides (data not shown). Finally, the shift in the n-Src loop required for MIP binding (Fig. 5) likely interferes with the binding of NS5A, further explaining the competition between the two peptides (Fig. 7).

These findings led us to consider earlier reports of protein–protein interactions via the SH3 domain and specifically whether there is other evidence of noncanonical interactions. Previous reports suggest that two proteins, HPK1 and GCK, interact with the SH3 domain of MLK3. However, as they were assumed to interact with the SH3 domain via their P*XX*P motifs, only proline-rich stretches were subjected to further analysis (42, 46). In the literature, only one known protein–protein interaction has been reported to occur with the SH3 domain of MLK3 through a noncanonical P*XX*P motif. The SH3 domain of MLK3 can bind a region located between the leucine zipper and CRIB motif in an intramolecular fashion, resulting in autoinhibition of the kinase domain (43). However, the exact site of this interaction on the SH3 domain still remains to be determined. Our discovery of a noncanonical binding site on the surface of MLK3 SH3 that can accept motifs devoid of any proline residues could shed new light on the mechanism of ligand binding to the SH3 domain of MLK3. Based on findings of phage-displayed peptide ligands corresponding to cellular proteins (5), we suspect that a cellular protein exists that binds the MLK SH3 domain in a manner equivalent to MIP and are actively attempting to discover it. Finally, the presence of a unique binding site makes MLK3 SH3 even more attractive as a potential drug target, offering a new site for design or discovery of a specific inhibitor.

## Experimental procedures

### Protein expression and purification for affinity selections and ELISAs

Two plasmids, gifts from Dr. Gerardo Morfini, University of Illinois at Chicago, encoding a GST protein fused to either the WT or mutant Y52A of the SH3 domain of MLK3 (UniProt entry Q16584; aa 43–104), respectively, between BamHI and EcoRI sites, were transformed into the *Escherichia coli* strain Rosetta(DE3)pLysS. DNA fragments encoding the SH3 domain of MLK1 (UniProt entry P80192; aa 52–116), MLK2 (UniProt entry Q02779; aa 16–81), and MLK4 (UniProt entry Q5TCX8; aa 38–102) were commercially prepared as gBlocks® Gene Fragments (Integrated DNA Technologies, Inc.). All coding regions were codon-optimized for *E. coli* expression. DNA fragments were cleaved with BamHI-HF® and EcoRI-HF® (New England Biolabs) and subsequently ligated into pGex-2T vector. The resulting DNA constructs, pGex-MLK1 SH3, pGex-MLK2 SH3, and pGex-MLK4 SH3 (verified by sequencing) were transformed into *E. coli* strain BL21(DE3).

For all constructs, the cells were initially grown at 37 °C in 2× YT medium (16 g of tryptone, 10 g of yeast extract, 5 g of NaCl/liter), which was supplemented with 50 μg/ml carbenicillin (CB) and 12.5 μg/ml chloramphenicol, and then diluted (1:100) into fresh 2× YT medium supplemented only with 50 μg/ml CB. Protein expression was induced with 0.5 mm isopropyl β-d-thiogalactopyranoside (IPTG) when the culture reached an *A*_600 nm_ of 0.6, and cells were incubated overnight at 27 °C at 250 rpm. Cells were harvested by centrifugation, and the pellets were resuspended in 1× bind/wash buffer (Novagen), which was supplemented with cOmplete^TM^ EDTA-free Protease Inhibitor Mixture (Roche Applied Science), and then lysed by sonication. Subsequent purification steps were carried out using GST·Bind^TM^ resin (Novagen) following the manufacturer's protocol. Following the elution step, the proteins were exchanged into 20 mm Tris, 150 mm NaCl, pH 8.0, using Zeba^TM^ Spin Desalting Columns (Thermo Fisher Scientific, Inc.) and then concentrated with Amicon® Ultra centrifugal filters (EMD Millipore) and stored at −80 °C. To confirm the purity, the samples were resolved by SDS-PAGE and detected with Coomassie Brilliant Blue staining.

### Isolation of peptide ligand to the SH3 domain of MLK3

Screens for peptide ligands were performed following a protocol published previously (11). The purified target protein, GST-MLK3 SH3-WT, was prebound to GSH magnetic beads (Pierce, catalogue number 88821), and nonspecific binding sites were blocked with phosphate-buffered saline (PBS; 137 mm NaCl, 3 mm KCl, 8 mm Na_2_HPO_4_, 1.5 mm KH_2_PO_4_) containing 3% (w/v) bovine serum albumin (BSA). To isolate potential peptide ligands, the targets were incubated with a 12-mer combinatorial phage-displayed peptide library, ANL7 (51). After three rounds of affinity selection, individual phage clones were isolated, and their binding was evaluated via phage ELISA (11). Positive clones were then sequenced.

### Sequence analysis of phage-displayed MIP

To evaluate the phage-displayed MIP (NH_2_-AIRINPNGTWSRQAETVES-COOH; insert (underlined) + linker region), isolated from ANL7 library (51), both WT and modified sequences (see Fig. S2) were cloned into a phage display vector, SAM (51), as an N-terminal fusion to the pIII capsid protein of M13 bacteriophage. All constructs were generated via Kunkel mutagenesis, described in detail elsewhere (11), and facilitated type 3 (pentavalent) display. Phage-displayed recombinants were evaluated for their binding to GST-MLK3 SH3 via phage ELISA as described elsewhere (11).

### Alanine scanning of phage-displayed MLK3 MIP

All constructs were generated via Kunkel mutagenesis (11, 57, 58) and facilitated type 3 (pentavalent) display. The single-stranded DNA template was generated using a modified bacteriophage M13 vector (SAM) containing an amber stop codon (TAG) within the linker upstream of the N terminus of pIII (51). The oligonucleotides used for generation of all clones were synthesized by Integrated DNA Technologies, Inc. and are shown in Table S1. All constructs were verified by DNA sequencing, and phage clones were evaluated for their binding to the target (GST-MLK3 SH3) via phage ELISA (11). A truncated version of MIP (ΔA1) was used as a negative control (see Fig. S2).

### Peptide synthesis

All peptides were synthesized at the Research Resource Center, University of Illinois at Chicago, and were of >90% purity as verified by MS. Two versions of MIP, unlabeled (AIRINPNGTWSRQAETVES) and biotinylated at the C terminus (AIRINPNGTWSRQAETVES-K-biotin), were used to determine the peptide's IC_50_. Two versions of NS5A-derived peptide (UniProt entry W8GG88; aa 2320–2331), unlabeled (KKAPTPPPRRRR-GGG) and biotinylated at the C terminus (KKAPTPPPRRRR-GGG-K-biotin), were used to determine the peptide's IC_50_. The biotinylated NS5A and unlabeled MIP peptide were used in competition ELISA. The biotinylated MIP, MIP-R12A (AIRINPNGTWSAQAETVES-K-biotin; as negative control), and NS5A peptide were used in specificity ELISA to determine whether MIP and NS5A can cross-react with MLK1–4.

### IC_50_ determination for MIP binding to the MLK3 SH3 domain

To determine the IC_50_ value by competition, the biotinylated form of MIP (AIRINPNGTWSRQAETVES-K-biotin; 100 μl, ≈2 μm) was captured in wells of a Nunc MaxiSorp^TM^ flat-bottom 96-well microtiter plate (Thermo Fisher Scientific), which had been previously coated with NeutrAvidin^TM^ (100 μl, 7.5 μg/ml; Thermo Fisher Scientific) and blocked with 3% skim milk in PBS. Separately, the GST-MLK3 SH3 fusion protein (5 μg/ml) was preincubated (1–1.5 h) with increasing concentrations (≈0.01–100 μm) of unlabeled MIP (AIRINPNGTWSRQAETVES) or unlabeled NS5A (KKAPTPPPRRRR-GGG-K) as competitor and then allowed to interact for 45 min with biotinylated MIP prebound on the ELISA plate. After three washes with PBS containing 0.1% Tween 20 (PBST), retention of the SH3 domain fusion protein in wells was detected with an anti-GST antibody conjugated to horseradish peroxidase (HRP) (GE Healthcare; 45 min; 1:5000 in PBST). Following three washes with PBST, the chromogenic substrate solution 2,2-azinobis(3-ethylbenzthiazoline-6-sulfonic acid) (ABTS; Pierce); 10 ml of 50 mm sodium citrate, pH 4.0; and 100 μl of 3% H_2_O_2_ were added to the plate (100 μl/well). The absorbance was measured at 405 nm with a POLARstar OPTIMA microtiter plate reader (BMG Labtech). This process was then repeated by testing the binding of the GST-MLK3 SH3 fusion protein to immobilized NS5A (KKAPTPPPRRRR-GGG-K-biotin; 100 μl, ≈2 μm) in the presence of unlabeled MIP or NS5A competitor.

### Gene synthesis, cloning, protein expression, and purification for structural analysis

Two DNA fragments encoding the SH3 domain of MLK3 (UniProt entry Q16584), MLK3 SH3(41–105) and MLK3 SH3(44–103), and four fragments encoding SH3 domain-MIP fusions, MLK3 SH3(41–105)-MIP(1–19), MLK3 SH3(41–105)-MIP(1–13), MLK3 SH3(41–105)-MIP(1–13)W10A, and MLK3 SH3(41–105)-MIP(1–13)R12A, were commercially prepared as gBlocks Gene Fragments. One fragment encoding SH3 domain fused to an NS5A (UniProt entry A0A076MER2) peptide, MLK3 SH3(41–105)-NS5A(2325–2332), was created by amplifying MLK3 SH3(41–105) with T7 promoter primer and a reverse primer (see Table S1). The subsequent insert contained MLK3 SH3(41–105) with the NS5A fragment (aa 2325–2332) at the C terminus. All coding regions were codon-optimized for *E. coli* expression. DNA fragments were cleaved with NdeI and BamHI-HF (New England Biolabs) and subsequently ligated into a modified version of the pET14b expression vector where the N-terminal His_6_ tag is followed by a SUMO protease cleavage site. The resulting DNA constructs (verified by sequencing) were transformed into *E. coli* strain BL21 C41(DE3). Cells were grown at 37 °C in 2× YT medium, which was supplemented with either 50 μg/ml CB or 100 μg/ml ampicillin, protein expression was induced with 0.1–0.3 mm IPTG at an *A*_600 nm_ of 0.6–0.7, and cells were cultured overnight at 22 °C. Cells were harvested by centrifugation; washed with 200 mm KCl, 50 mm Tris, pH 7.4; and lysed by sonication in 25 mm Tris, pH 7.4, 200 mm KCl, 10 mm MgCl_2_, 10% glycerol, 1% Triton X-100, 1 mm phenylmethylsulfonyl fluoride (PMSF). Clarified supernatant was loaded onto a 5-ml HisTrap^TM^ HP Ni-Sepharose column (GE Healthcare), and the column was washed with 25 mm Tris, pH 7.4, 500 mm NaCl supplemented with 10 and 25 mm imidazole. Protein was eluted in the same buffer, which was supplemented with 250 and 500 mm imidazole, and the fractions were pooled together. The His_6_-SUMO tag was cleaved with SUMO protease while dialyzing against 25 mm Tris, pH 7.4, 500 mm NaCl, 10 mm imidazole, and the tag was removed by loading the sample back onto a nickel column. Collected fractions containing purified protein were concentrated and injected onto an S-200 size-exclusion column (GE Healthcare) equilibrated with 25 mm Tris-HCl, pH 7.4, 500 mm NaCl. To confirm the purity, collected samples were analyzed by SDS-PAGE and detected with Coomassie Brilliant Blue staining. All purified proteins were concentrated and stored at −80 °C.

### Crystallization

All crystals were grown at 20 °C using the hanging-drop vapor-diffusion method. Crystals of MLK3 SH3(41–105)-MIP(1–19) were grown using a reservoir condition containing 0.1 m NaH_2_PO_4_, 0.1 m KH_2_PO_4_, 0.1 m MES, pH 6.5, 1.5 m NaCl. Drops of 4 μl were set up at a 1:1 ratio of protein (9 mg/ml) to reservoir. These crystals grew as long pyramids, ∼250 μm in length and 50 μm at the base. Prior to data collection, crystals of MLK3 SH3(41–105)-MIP(1–19) were soaked in the reservoir solution containing 30% ethylene glycol for cryoprotection. To grow crystals of MLK3 SH3(44–103), drops of 3 and 4 μl were set up, respectively, at 2:1 and 1:1 ratios of protein (15.7 mg/ml) to reservoir solution (1.4 m sodium malonate, pH 6.5). Cube-shaped crystals appeared within 2–3 days. Prior to data collection, crystals of MLK3 SH3(44–103) were soaked in mother liquor with 30% ethylene glycol as cryoprotectant.

Crystals of MLK3 SH3(44–103)-NS5A(2325–2332) at 3.6 mg/ml were set up in 2-μl drops at a 1:1 ratio with a mother liquor of 0.1 m KH_2_PO_4_, 0.1 m NaH_2_PO_4_, 0.1 m MES, pH 6.5, 2.5 m NaCl. Crystals grew over approximately 2 months and developed as clusters of square pyramids. Crystals were cryopreserved prior to data collection with 30% glycerol.

### Data collection and structure determination of apo, MIP-, and NS5A-bound MLK3 SH3

Diffraction data for MLK3 SH3–MIP, MLK3 SH3–NS5A, and apo MLK3 SH3 were obtained at the Life Sciences Collaborative Access Team ID beamline at the Advanced Photon Source, Argonne National Laboratory (wavelength, 0.979 Å; temperature, 100 K) (refer to Table 1 for data collection and refinement statistics). Data processing was executed using XDS (59). The structure of MLK3 SH3–MIP was solved by molecular replacement using MOLREP (60) with the available structure of the MAP3K10 SH3 domain (PBD code 2RF0) as the search model. Likewise, the apo MLK3 SH3 and MLK3 SH3–NS5A structures were solved using MOLREP using the MIP-bound structure as the model. Refinement was accomplished with REFMAC5 (61). Because the MLK3 SH3–NS5A data indicated the C2 space group with a unique β angle close to 120° (*i.e.* 120.006°), we also analyzed the data using phenix.xtriage, which confirmed C2 as the correct choice of space group (62). All structural figures were generated with MacPyMOL (PyMOL^TM^ Molecular Graphics System, version 1.6, Schrödinger, LLC).

### Analytical size-exclusion chromatography

To determine oligomeric state of several MLK3 SH3 fusion proteins, samples were injected into a Superdex 200 10/300 GL size-exclusion chromatography column (GE Healthcare) in two independent experiments. Two samples, MLK3 SH3(41–105)-MIP(1–19) and MLK3 SH3(41–105), at 2 mg of protein/200-μl injection, were analyzed in the first experiment (Fig. S3). Three additional samples of MLK3 SH3 domain-MIP fusion proteins were analyzed in a second experiment by injecting 750 μg of protein in 200 μl: MLK3 SH3(41–105)-MIP(1–13), MLK3 SH3(41–105)-MIP(1–13)W10A, and MLK3 SH3(41–105)-MIP(1–13)R12A (Fig. S4). Prior to the injections, the column was equilibrated with 25 mm Tris-HCl, pH 7.4, 500 mm NaCl.

### Competitive ELISA to determine competition between MIP and NS5A

Competition ELISA was performed to determine whether MIP and NS5A peptides compete in binding the MLK3 SH3 domain and to calculate the relative binding strength (*i.e.* IC_50_) of the NS5A peptide. Biotinylated NS5A peptide (KKAPTPPPRRRR-GGG-K-biotin; 100 μl, ≈ 2 μm) was captured in wells of a Nunc MaxiSorp flat-bottom 96-well plate, which had been coated with NeutrAvidin (100 μl, 7.5 μg/ml), and blocked with 3% skim milk in PBS. Separately, the GST-MLK3 SH3(43–104) domain fusion protein (5 μg/ml) was preincubated (1–1.5 h) with increasing concentration (0.02–100 μm) of unlabeled MIP as competitor and then allowed to interact for 1 h with biotinylated MIP prebound on the ELISA plate (Fig. S7). The assay was then completed as described above for IC_50_ determination of MIP.

### Specificity ELISA to determine MIP and NS5A peptide binding to other members of MLK subfamily

To determine whether MIP and NS5A can cross-react with other members of the MLK subfamily, SH3 domains of MLK1, MLK2, and MLK4 were purified as GST fusion proteins as described above. To determine their binding properties, 100 μl (≈2 μm) of biotinylated peptides, MIP (AIRINPNGTWSRQAETVES-K-biotin), MIP-R12A (AIRINPNGTWSAQAETVES-K-biotin; as negative control), and NS5A (KKAPTPPPRRRR-GGG-K-biotin), were first captured in wells of a Nunc MaxiSorp flat-bottom 96-well plate coated with NeutrAvidin (100 μl, 7.5 μg/ml) and then blocked with 2% skim milk in PBS. The GST-MLK1 SH3, GST-MLK2 SH3, GST-MLK2 SH3, and GST-MLK4 SH3 domain fusion proteins (0.15 μm) were incubated with each of the target peptides, MIP, NS5A, or MIP-R12A, for 1–1.5 h. The rest of the assay was performed as described above for IC_50_ determination of MIP.

### Data resources

The coordinates for apo SH3, SH3–MIP complex, and SH3–NS5A complex have been deposited in the PDB under accession codes 5K28, 5K26, and 6AQB, respectively.

## Author contributions

M. E. K. and S. L. K. formal analysis; M. E. K. and J. E. M. validation; M. E. K., S. L. K., S. K., and J. E. M. investigation; M. E. K., S. L. K., A. L., and B. K. K. writing-original draft; M. E. K., S. L. K., S. K., J. E. M., A. L., and B. K. K. writing-review and editing; S. L. K. data curation; S. L. K. visualization; S. K., A. L., and B. K. K. resources; A. L. and B. K. K. conceptualization; A. L. and B. K. K. supervision; A. L. project administration; B. K. K. funding acquisition.

## Supplementary Material

Supporting Information

## Footnotes

3 The abbreviations used are:

SH3: Src homology 3
MLK: mixed-lineage kinase
MIP: MLK3 SH3–interacting peptide
NS5A: nonstructural 5A
r.m.s.d.: root mean square deviation
PDB: Protein Data Bank
JNK: c-Jun N-terminal kinase
ERK: extracellular signal-regulated kinase
CRIB: Cdc42/Rac interactive binding
HCV: hepatitis C virus
HPK1: hematopoietic progenitor kinase 1
GCK: germline center kinase
GST: GSH *S*-transferase
aa: amino acids
CB: carbenicillin
IPTG: isopropyl β-d-thiogalactopyranoside
HRP: horseradish peroxidase
SUMO: small ubiquitin-like modifier.
