# Supplementary material for: Identification of two distinct peptide-binding pockets in the SH3 domain of human mixed-lineage kinase 3
Source: J Biol Chem. 2018 Jul 6;293(35):13553–65. doi: 10.1074/jbc.RA117.000262 (PMC6120190; doi:10.1074/jbc.RA117.000262)
Supplement: Supporting Information [file supp_293_35_13553__index.html]

Identification of two distinct peptide-binding pockets in the SH3 domain of human mixed-lineage kinase 3 — Peptide ligand for the MLK3 SH3 domain — Identification of two distinct peptide-binding pockets in the SH3 domain of human mixed-lineage kinase 3 — Peptide ligands for the MLK3 SH3 domain — Supporting Information 

# Identification of two distinct peptide-binding pockets in the SH3 domain of human mixed-lineage kinase 3

## Supporting Information

- Supplementary figures and tables. - Supplementary figures and tables
- Supporting Data Table 1 - Supporting Data Table 1
