# Supplementary material for: Identification of two distinct peptide-binding pockets in the SH3 domain of human mixed-lineage kinase 3
Source: J Biol Chem. 2018 Jul 6;293(35):13553–65. doi: 10.1074/jbc.RA117.000262 (PMC6120190; doi:10.1074/jbc.RA117.000262)
Supplement: Supporting Information [file supp_RA117.000262_132892_2_supp_160580_pmzs5f.docx]

**Identification of two competing binding pockets in the**

**SH3 domain of human MLK3 kinase**

Malgorzata E. Kokoszka, Stefanie L. Kall, Sehar Khosla, Jennifer E. McGinnis,

Arnon Lavie, and Brian K. Kay

**Supplementary Materials**

**Figure S1-** SDS-PAGE of MLK3 SH3 domains

**Figure S2-** Truncation analysis of phage-generated peptide.

**Figure S3-** Comparison of apo vs peptide-bound MLK3 SH3.

**Figure S4-** W10 and R12 in MIP are critical for intermolecular interaction with MLK3 SH3 *in vitro*.

**Figure S5-** Binding properties of synthetic NS5A peptide.

**Figure S6-** Structural alignment suggests MIP binding to be likely conserved across the MLKs.

**Figure S7-** Format of the competitive IC50 experiment.

**Table S1-** Primers used for alanine scanning of MIP and generation of fusion constructs.


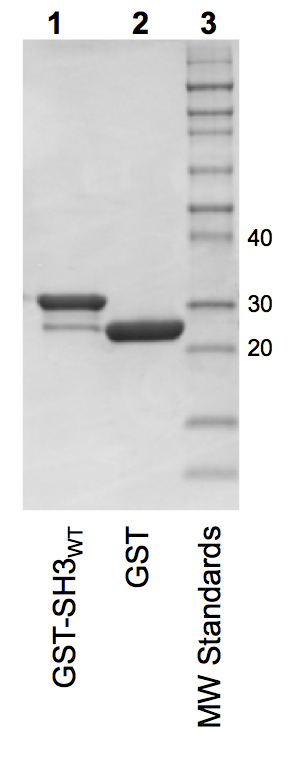


**Figure S1. SDS-PAGE of MLK3 SH3 domains.** SDS-PAGE analysis of WT (lane 1) form of the MLK3 SH3 domain fused to GST. Lane 2 represents GST protein alone. Lane 3 represents molecular weight (MW) standards in kDa.


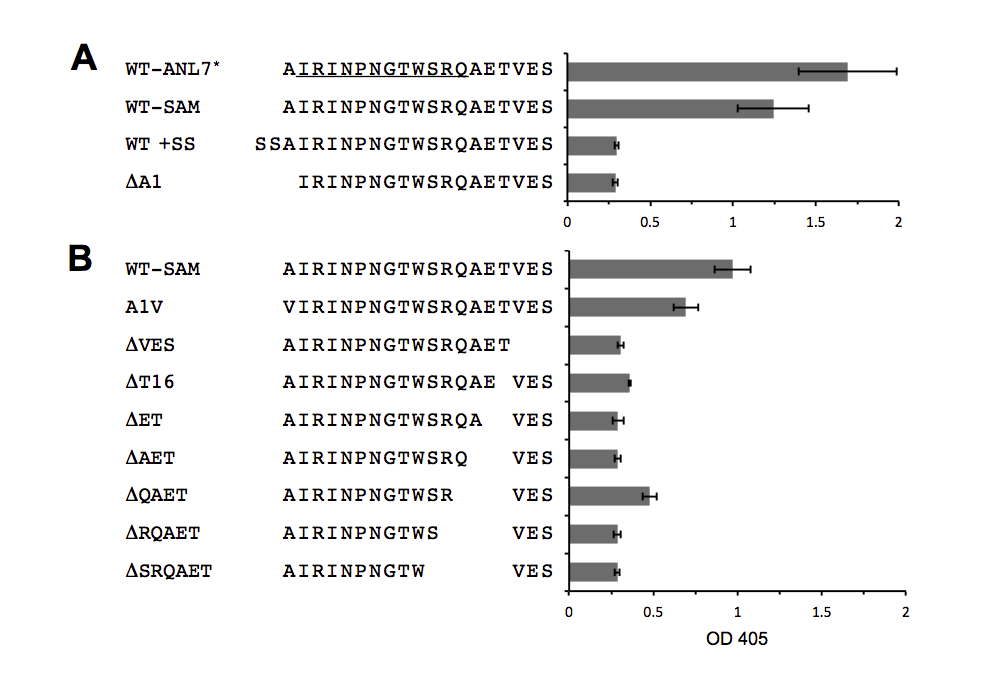


**Figure S2. Truncation analysis of phage-generated peptide.** WT-ANL7 represents original phage-displayed MLK3 SH3 ligand isolated from ANL7 library (1). To generate modified and truncated peptide sequences, desired variants were fused to the N- terminus of the pIII capsid protein of bacteriophage M13 using a phage display vector SAM (47), to facilitate type 3 pentavalent display. WT-SAM represents MIP ligand displayed using SAM. All phage-displayed variants were evaluated for their binding to MLK3 SH3 via phage ELISA, in two independent experiments corresponding to panel **A** and **B**. The experiments were performed in triplicate and the results are an averaged value. Error bars reflect the standard deviation of each triplicate measurement.


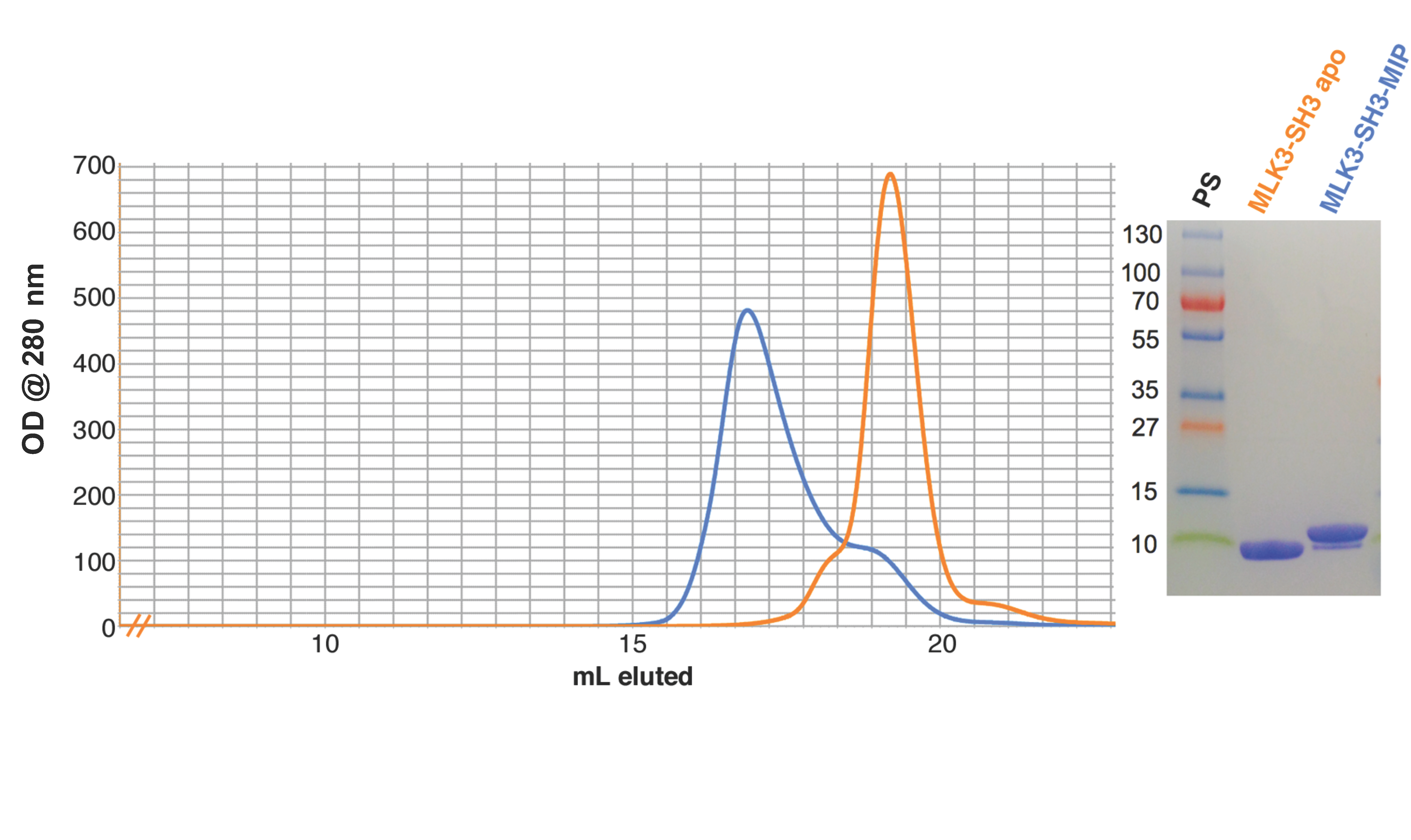


**Figure S3. Comparison of apo vs peptide-bound MLK3 SH3.** Gel filtration chromatography of MLK3 SH3 and MLK3 SH3-MIP(1-19) fusion using Superdex 200 10/300 GL. Both constructs were expressed and purified as His6-SUMO fusions and the tag cleaved with SUMO protease. Elution profile for SH3-MIP(1-19) fusion corresponds to at least twice the MW of the monomer, suggesting formation of a higher order oligomer mediated by the interaction of the MLK3 SH3 domain with the MIP region of a different monomer, suggesting that the molecular interaction occurs in *cis* rather than *trans*. SDS-PAGE shows both constructs corresponding to the correct sizes of 7.2 and 9.4 kDa, respectively. PS corresponds to MW standards (kDa).


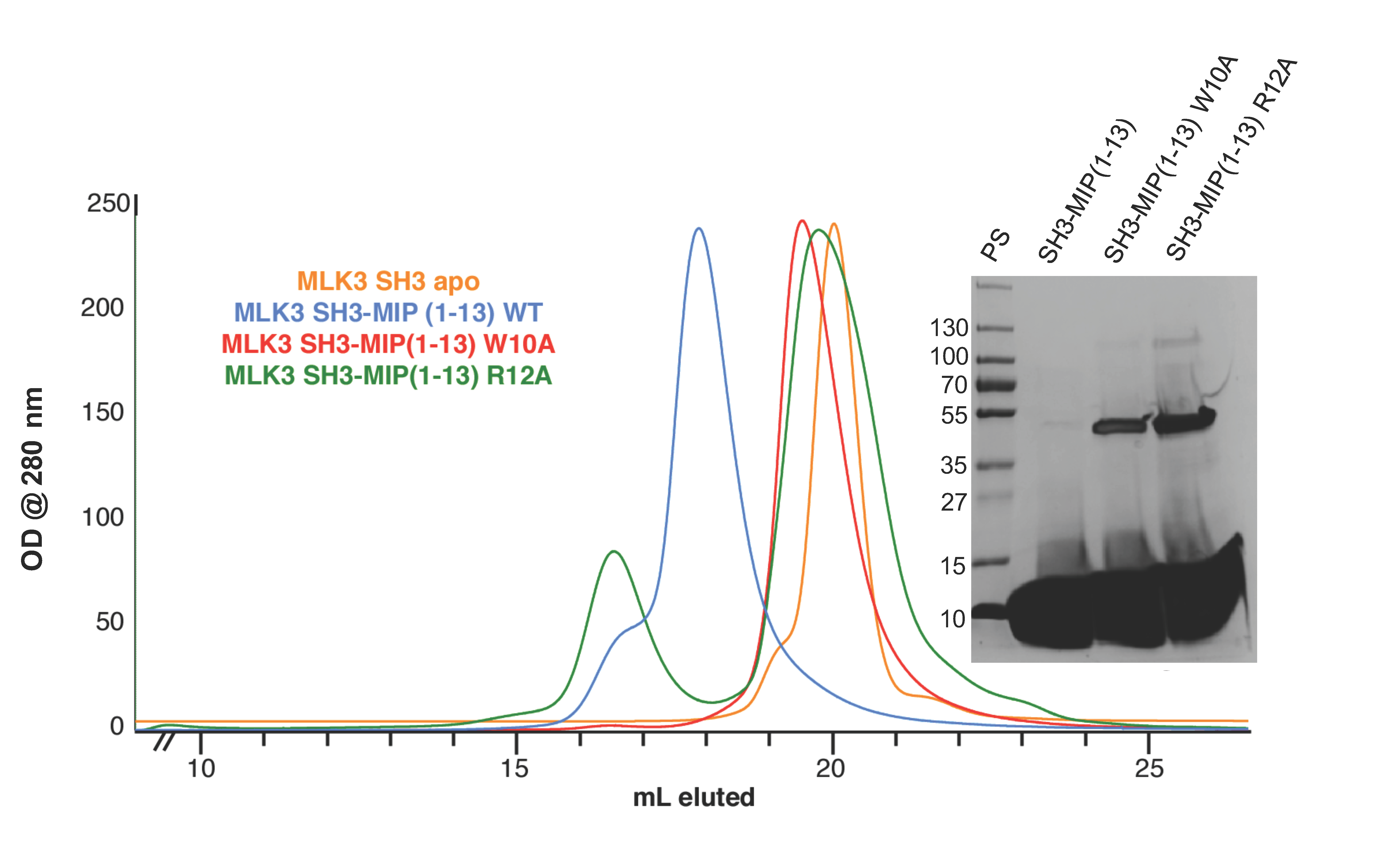


**Figure S4. W10 and R12 in MIP are critical for intermolecular interaction with MLK3 SH3 *in vitro*.** Three fusion proteins composed of MLK3 SH3(41-105) domain linked via its C-terminus to one of the MIP variant: MIP(1-13) (Blue), MIP(1-13)W10A (Red), or MIP(1-13)R12A (Green), and have been analyzed using gel filtration chromatography. Corresponding sequences of MIP variants are: AIRINPNGTWSRQ (Blue), AIRINPNGTASRQ (Red), and AIRINPNGTWSAQ (Green). As illustrated in Figure S3, elution profile for WT suggests the formation of a higher order oligomer. However, both W10A and R12A mutants elute at fraction corresponding to the size of a monomeric fusion protein, as judged by the peptide-free MLK3 SH3 (Orange). The chromatogram corresponding to MLK3 SH3 (Orange) was scaled down by approximately 33%. SDS-PAGE shows three constructs (WT, W10A, R12A) prior to injections corresponding to the correct size of a monomer (~8.7 kDa). Additional peak at elution volume around 16mL corresponds with contaminant of approximately 50 kDa visible on SDS-PAGE, co-purified along with the fusion proteins. PS corresponds to MW standards (kDa).

**
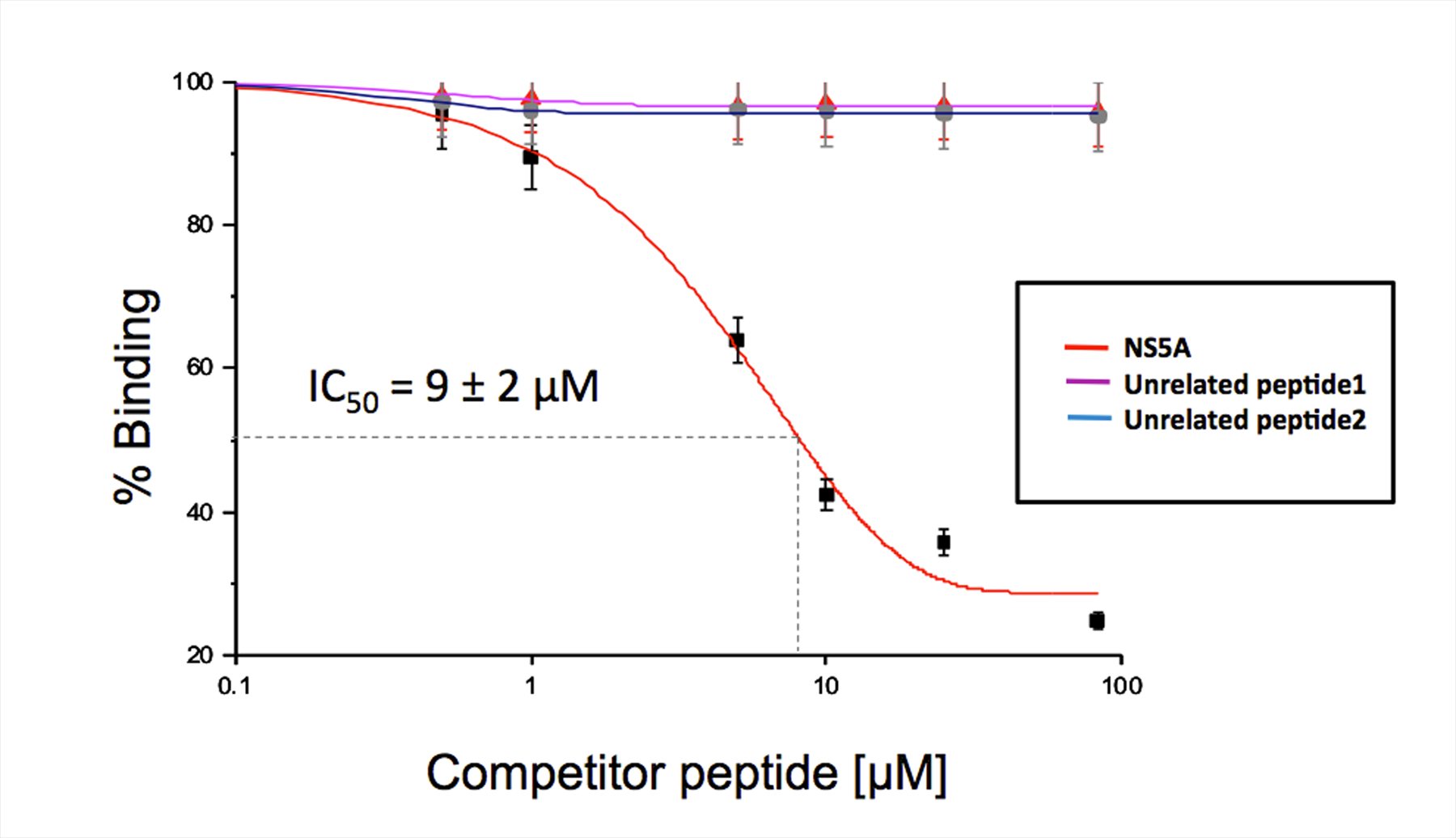
**

**Figure S5. Binding properties of synthetic NS5A peptide.** To determine the IC_50_ value of NS5A peptide, a GST-MLK3 SH3(43-104) domain fusion protein was pre- incubated with increasing concentration of unlabeled NS5A (KKAPTPPPRRRR-GGG) as competitor and then allowed to interact with biotinylated NS5A (KKAPTPPPRRRR- GGG-K-bio) immobilized on NeutrAvidin coated 96-well ELISA plate. Binding of the SH3 domain of MLK3 was detected with anti-GST antibody conjugated to HRP, and the levels presented as percentage of binding in the absence of competitor. The experiments were performed in triplicate and the results are averaged values. Error bars reflect the standard deviation of each triplicate measurement. Curve fitting was performed with OriginPro 9.1 software.


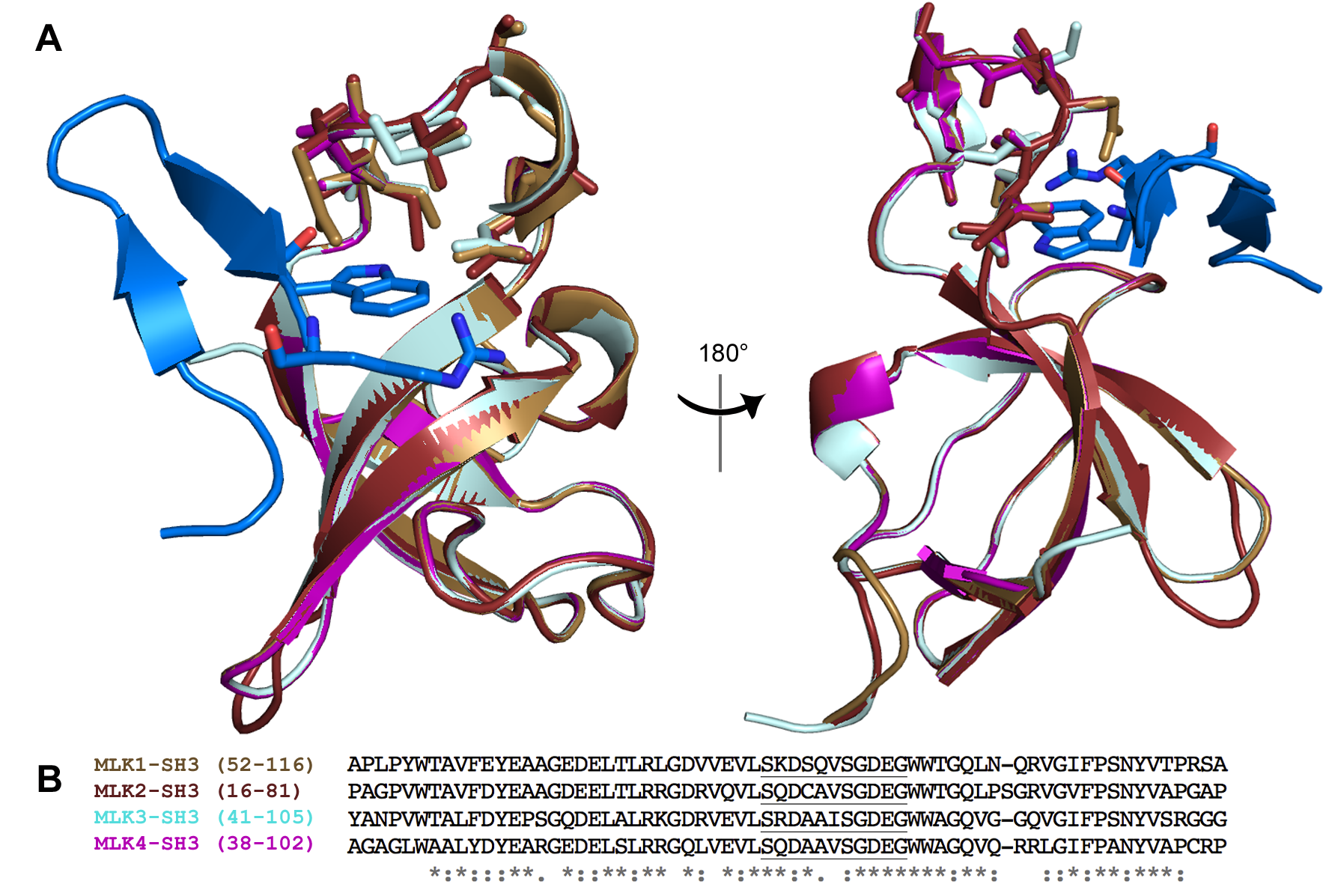


**Figure S6. Structural alignment suggests MIP binding to be likely conserved across the MLKs**. **A.** Structural protein homology models for SH3 domains of MLK1 (UniProt entry P80192, sand), MLK2 (UniProt entry Q02779, firebrick), and MLK4 (UniProt entry Q5TCX8, magenta) were generated by SWISS-MODEL (2-5), using solved structure of MLK3 SH3 (cyan) in complex with MIP (blue) as a template. The MIP-binding n-Src loop is shown in sticks. **B.** Sequence alignment of SH3 domains of MLK1 – MLK4 generated using ClustalW2 (6). Underlined is the loop sequence critical for binding the MIP, corresponding to the extended n-Src loop. Based on the sequence similarity and obtained 3D models, it is predicted that binding of MIP could be conserved across all the remaining members of the MLK subfamily.

**
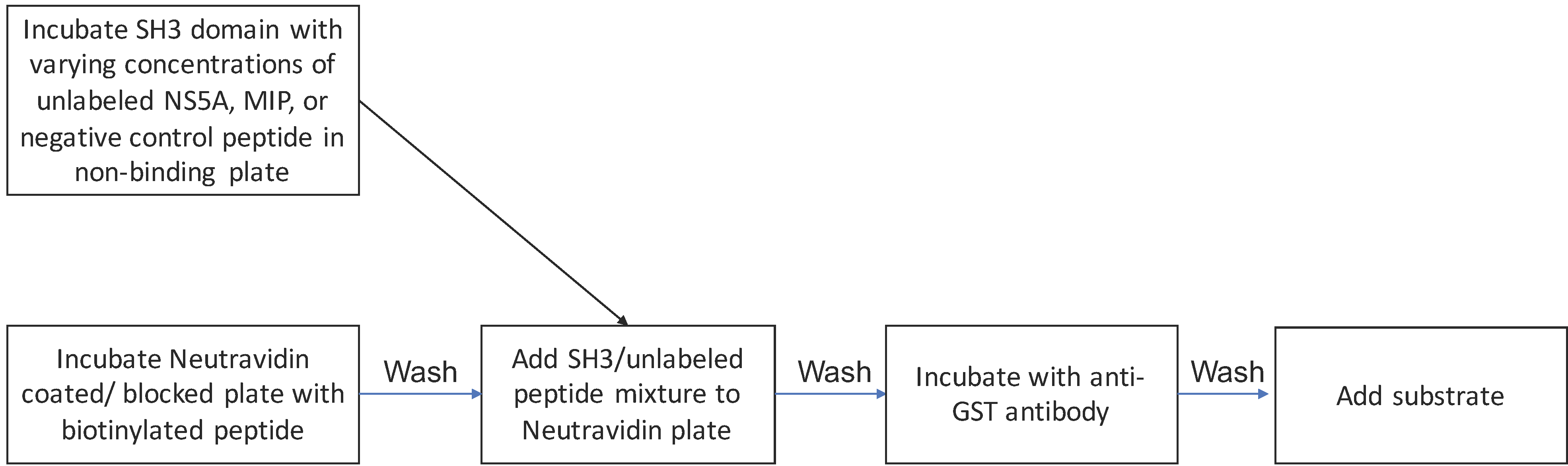
**

**Figure S7. Format of the competitive IC_50_ experiment.** The various steps in the experiments are described. A decrease in signal demonstrates that the unlabeled competitor is binding at the same site on the MLK3 SH3 domain as the biotinylated peptide.
