# Supplementary material for: Identification of two distinct peptide-binding pockets in the SH3 domain of human mixed-lineage kinase 3
Source: J Biol Chem. 2018 Jul 6;293(35):13553–65. doi: 10.1074/jbc.RA117.000262 (PMC6120190; doi:10.1074/jbc.RA117.000262)
Supplement: Supporting Information [file supp_RA117.000262_132892_2_supp_164822_pbg87n.docx]

**Table S1**. Primers used for alanine scanning of MIP and generation of fusion constructs.

|  | **Mutation** | **Primer (5’ – 3’)** |
| --- | --- | --- |
| **MIP** | **WT** | GTTGTTTCTTTCTATTCTCACTCCGCCATTCGTATTAATCCTAATGGTACTTGGTCGAGGCAGGCCGAAACTGTTGAAAGTTGTTTAGCAAAATCCC |
|  | **A1V** | GTTGTTTCTTTCTATTCTCACTCCGTTATTCGTATTAATCCTAATGGTACTTGGTCGAGGCAGGCCGAAACTGTTGAAAGTTGTTTAGCAAAATCCC |
|  | **I2A** | GTTTCTTTCTATTCTCACTCCGCCGCACGTATTAATCCTAATGGTACTTGGTCGAGGCAGGCCGAAACTGTTGAAAGTTG  TTTAGCAAAA |
|  | **R3A** | GTTTCTTTCTATTCTCACTCCGCAATTGCAATTAATCCTAATGGTACTTGGTCGAGGCAGGCCGAAACTGTTGAAAGTTG  TTTAGCAAAA |
|  | **I4A** | GTTTCTTTCTATTCTCACTCCGCAATTCGTGCAAATCCTAATGGTACTTGGTCGAGGCAGGCCGAAACTGTTGAAAGTTG  TTTAGCAAAA |
|  | **N5A** | GTTTCTTTCTATTCTCACTCCGCCATTCGTATTGCACCTAATGGTACTTGGTCGAGGCAGGCCGAAACTGTTGAAAGTTG  TTTAGCAAAA |
|  | **P6A** | GTTTCTTTCTATTCTCACTCCGCCATTCGTATTAATGCAAATGGTACTTGGTCGAGGCAGGCCGAAACTGTTGAAAGTTG  TTTAGCAAAA |
|  | **N7A** | GTTTCTTTCTATTCTCACTCCGCCATTCGTATTAATCCTGCAGGTACTTGGTCGAGGCAAGCCGAAACTGTTGAAAGTTG  TTTAGCAAAA |
|  | **G8A** | GTTTCTTTCTATTCTCACTCCGCCATTCGTATTAATCCTAATGCAACTTGGTCGAGGCAAGCCGAAACTGTTGAAAGTTG  TTTAGCAAAA |
|  | **T9A** | GTTTCTTTCTATTCTCACTCCGCAATTCGTATTAATCCTAATGGTGCATGGTCGAGGCAAGCCGAAACTGTTGAAAGTTG  TTTAGCAAAA |
|  | **W10A** | GTTTCTTTCTATTCTCACTCCGCAATTCGTATTAATCCTAATGGTACTGCATCGAGGCAAGCCGAAACTGTTGAAAGTTG  TTTAGCAAAA |
|  | **S11A** | GTTTCTTTCTATTCTCACTCCGCAATTCGTATTAATCCTAATGGTACTTGGGCAAGGCAAGCCGAAACTGTTGAAAGTTG  TTTAGCAAAA |
|  | **R12A** | GTTTCTTTCTATTCTCACTCCGCAATTCGTATTAATCCTAATGGTACTTGGTCTGCACAAGCCGAAACTGTTGAAAGTTG  TTTAGCAAAA |
|  | **Q13A** | GTTTCTTTCTATTCTCACTCCGCAATTCGTATTAATCCTAATGGTACTTGGTCTAGGGCAGCCGAAACTGTTGAAAGTTG  TTTAGCAAAA |
|  | **A14V** | GTTTCTTTCTATTCTCACTCCGCAATTCGTATTAATCCTAATGGTACTTGGTCTAGGCAGGTTGAAACTGTTGAAAGTTG  TTTAGCAAAA |
|  | **E15A** | GTTTCTTTCTATTCTCACTCCGCAATTCGTATTAATCCTAATGGTACTTGGTCTAGGCAGGCAGCAACAGTTGAAAGTTG  TTTAGCAAAA |
|  | **T16A** | GTTTCTTTCTATTCTCACTCCGCAATTCGTATTAATCCTAATGGTACTTGGTCTAGGCAGGCAGAAGCAGTTGAAAGTTG  TTTAGCAAAA |
|  | **V17A** | GTTTCTTTCTATTCTCACTCCGCAATTCGTATTAATCCTAATGGTACTTGGTCTAGGCAGGCAGAAACTGCAGAAAGTTG  TTTAGCAAAATCC |
|  | **E18A** | TTGTTTCTTTCTATTCTCACTCCGCAATTCGTATTAATCCTAATGGTACTTGGTCTAGGCAGGCAGAAACTGTTGCAAGT  TGTTTAGCAAAATCC |
|  | **S19A** | TTGTTTCTTTCTATTCTCACTCCGCAATTCGTATTAATCCTAATGGTACTTGGTCTAGGCAGGCAGAAACTGTTGAAGCA  TGTTTAGCAAAATCCCAT |
| **SH3-NS5A** | **NS5A**  **(2325-2332)** | CGCGGATCCTTAGCGGCGAGGCGGAATAGGTGGTGCACCTCCTCCGCGGC |
